# Effectiveness of implementing the new bundles of surgical site infection (SSI) of *Sectio Caesarea* (SC) in post SC operative patients on the SSI incident rate

**DOI:** 10.1017/ash.2025.110

**Published:** 2025-09-03

**Authors:** Siti Jubaedah, Jajang Jamaludin, Elis Ecin, Tita Setiawati, Riyadi Adrizain

**Affiliations:** 1 Dr. Hasan Sadikin General Hospital, Bandung, West Java; 2 Al Ihsan Hospital, Bandung, West Java

**Keywords:** Bundles, surgical site infection, post *sectio caesarea* operation patient

## Abstract

**Background:** SSI cases in patients undergoing SC procedures are still a problem. Routine bundles are being implemented but infections still occur. So, it is necessary to evaluate existing bundles. **Research objective:** To compare the incidence of post-SC SSIs between the implementation of routine bundles in hospitals with new SSI bundles. **Method:** Quantitative research using a prospective cohort method on pregnant women of childbearing age (WCA) aged 15-49 years who were treated in the Dr. Hasan Sadikin General Hospital, Bandung. Data collection was carried out in the period October- November 2023 where SC was carried out. The sampling technique that will be used in this research is a non-probability sampling technique with a consecutive sampling method. The instruments used are the SSI indicator profile, hospital standard tool bundles, and new SSI tool bundles. Data analysis used the Fisher Exact test to compare the two groups and the efficacy formula to calculate the effectiveness of implementing the two bundles. **Research Results:** There were 710 cases of SC, and in 60 research samples in both groups for one month, the results of the intervention group with new bundles of SC showed 100% effectiveness with no cases or 0% during the 0-30 days post-SC operation monitoring period while the control group with hospital procedure bundles had one case or 3.3%. The Fisher Exact test analysis showed no significant difference (P> 0.005) between the intervention group with new SC bundles and the control group with hospital procedure bundles (P=1.000). **Conclusion:** The implementation of the new SSI bundles in the intervention group was proven to be effective with no cases of SSI when compared to the control group with hospital procedure bundles which contained one case of SSI.

